# Adversarial attack of sequence-free enhancer prediction identifies chromatin architecture

**DOI:** 10.1093/bioinformatics/btaf371

**Published:** 2025-06-24

**Authors:** Jamil Gafur, Olivia W Lang, William K M Lai

**Affiliations:** Department of Computer Science, University of Iowa, Iowa City, IA 52242, United States; Department of Molecular Biology and Genetics, Cornell University, Ithaca, NY 14850, United States; Department of Molecular Biology and Genetics, Cornell University, Ithaca, NY 14850, United States; Department of Computational Biology, Cornell University, Ithaca, NY 14850, United States

## Abstract

**Motivation:**

The wide range of cellular complexity created by multicellular organisms is due in large part to the intricate and synergistic interplay of regulatory complexes throughout the eukaryotic genome. These regulatory elements “enhance” specific gene programs and have been shown to operate in diverse networks that are distinct across cell states of the same organism. Attempts to characterize and predict enhancers have typically focused on leveraging information-dense DNA sequence in parallel with epigenomic assays. We examined the viability of enhancer prediction using only a minimal set of epigenomic datasets without direct DNA information.

**Results:**

We demonstrate that chromatin datasets are sufficient to identify enhancers genome-wide with high accuracy. By training networks leveraging data from multiple cell types simultaneously, we generated a cell-type invariant enhancer prediction platform that utilized only the patterns of protein binding for inference. We also showed the utility of swarm-based adversarial attacks [adversarial particle swarm optimization (APSO)] to deconvolute trained genomic neural networks for the first time. Critically, unlike saliency mapping or other game-theory based approaches, APSO is completely network-architecture independent and can be applied to any prediction engine to derive the features that drive inference.

**Availability and implementation:**

All software and code for data downloading, processing, enhancer inference, eXplainable AI (XAI), and complete figure generation are publicly available on GitHub at https://github.com/EpiGenomicsCode/ChromEnhancer and Zenodo at https://doi.org/10.5281/zenodo.15652797.

## 1 Introduction

The vast majority (>98%) of DNA in the human genome is composed of regulatory elements that do not code for protein-coding genes. These regulatory regions are biochemically active and intricately tied to diverse biological processes including gene transcription, repression, three-dimensional architecture, and many others (ENCODE 2012). Regulatory genomic loci that amplify gene expression, often irrespective of their precise location within the genome, are classified as “enhancers.” The complex combinatorial interactions between enhancers and their target genes are believed to be responsible in part for how complex multicellular organisms achieve distinct cell types even with identical DNA content in all cells of a single organism (Heinz *et al.* 2015, Choi *et al.* 2021).

Decades of genetic research has shown that many DNA mutations (i.e. single nucleotide polymorphisms, copy number variations, etc.) that are correlated to disease (i.e. cancer) are located outside the regions of the genome that code for genes (Zhang and Lupski 2015, Schipper and Posthuma 2022). It is suspected that many of these mutations are located within putative enhancer regions and may be indirectly perturbing gene expression by disrupting critical regulatory processes that in turn result in a disease state (Zaugg *et al.* 2022). This makes them prime candidates for identification and characterization to better understand the broad and multidimensional regulatory systems modulating our genomes. However, enhancer identification has been confounded by their apparent lack of universal DNA-sequence and their highly heterogeneous utilization across the broad spectrum of cell states within a multi-cellular organism.

While the sequence content of enhancers can vary site to site, it is generally accepted that enhancer activity is orchestrated by the proteins that bind them, defined here as the “epigenome” (Sahu *et al.* 2022). These protein complexes can be immunopurified from the genome, and the DNA attached to them can be sequenced to identify the exact region from which they originated (i.e. ChIP-seq) (Park 2009). This assay has been applied to thousands of targets in hundreds of cell lines, identifying common and unique features to enhancers in different cell types (ENCODE 2012, Lai *et al.* 2021). The common epigenomic features of enhancers include the presence of specific histone-tail post-translational modifications (e.g. H3K27ac and H3K4me1), protein binding events (i.e. p300) and biochemical activities like transcription (Calo and Wysocka 2013, Raisner *et al.* 2018, Tippens *et al.* 2020).

Previous deep-learning approaches have combined DNA-sequence and biochemical assays measuring protein-DNA (“chromatin”) interactions to great success at predicting enhancers genome-wide in distinct cell types (Min *et al.* 2017, Chen *et al.* 2021). However, model reliance on DNA sequence is potentially problematic as DNA is notorious for becoming progressively more mutated and distinct from the reference genome as cancer progresses (Martínez-Jiménez *et al.* 2020). Enhancers in particular are known to mutate in cancer, causing dramatic changes in cell identity (Kron *et al.* 2014). This combined with challenges in understanding how these “black box” networks use input data to make inference limits their potential clinical utility.

eXplainable AI (XAI) has developed numerous approaches to better understand and deconvolute the decision-making process of neural networks. Saliency was developed to understand 2D convolutional networks by calculating the gradient of activation as a function of any given input and has been adopted in genomics (Simonyan *et al.* 2013, Talukder *et al.* 2021). Similarly, additional approaches such as integrated gradient calculation and game theory-based approaches such as Shapley Additive exPlanations (SHAP) are now applied to networks trained on genomics and epigenomic datasets (Lundberg and Lee 2017, Sundararajan *et al.* 2017, Jha *et al.* 2020, Majdandzic *et al.* 2023). However, each of these techniques rely on the existence of “holdout” data to examine the features the trained network finds useful. We recently developed a novel XAI technique based on classic particle swarm optimization that complements existing XAI approaches by removing the requirement for hold-out data to apply XAI approaches (Gafur *et al.* 2024).

Here we describe an enhancer-prediction framework that uses only chromatin datasets (i.e. ChIP-seq) for accurate prediction and, in doing so, circumvents the requirement for parallel DNA-sequence information for inference. We demonstrate that a diverse range of network architectures can accurately predict enhancers genome-wide irrespective of specific cell type using a limited number of input training datasets. Additionally, we establish our adversarial particle swarm optimization (APSO) as a viable approach for characterizing what neural networks have learned with no *a priori* architectural knowledge. We characterize and confirm cell line-independent common chromatin features at enhancers. Critically, unlike previous annotation approaches (e.g. chromHMM, Segway, etc.), we demonstrate APSO’s ability to identify complex and combinatorial chromatin features of interest without any *a priori* annotation information by applying it to a large-scale network trained with hundreds of unique chromatin datasets (Ernst and Kellis 2012, Hoffman *et al.* 2012).

## 2 Methods

### 2.1 Data acquisition

All starting datasets were downloaded from the ENCODE data portal (Luo *et al.* 2020). These include Assay for Transposase-Accessible Chromatin (ATAC-seq) peak coordinates from ENCODE and STARR-seq peaks from alignment data for each of the four cell lines examined in this study (A549, HepG2, K562, and MCF-7). Alignment data for two replicate datasets of each of five different ChIP-seq targets (H3K4me1, H3K4me3, H3K27ac, H3K27me3, H3K36me3, CTCF, p300, and POLR2A) were downloaded across each cell line for the chromosome and cell line hold-out datasets. PRO-seq and GRO-seq FASTQ data were downloaded from SRA and aligned to the hg38 genome according to the reported best practices (Mahat *et al.* 2016). Run-on occupancy for each set of enhancer and random coordinates was calculated using the 5′ end of Read 1 with tags set to be equal across datasets using the ScriptManager analysis platform (Lang *et al.* 2022). Totally, 330 K562 ChIP-seq sequence alignment (BAM) files were downloaded from the ENCODE portal and used for the large network training. All data were on the hg38 genome build. The full list of accession codes is available in Table 1, available as supplementary data at *Bioinformatics* online.

### 2.2 Training and validation dataset generation

The performance of a neural network depends on the quality of the training and validation data, adhering to the principle of “garbage in, garbage out” (Kilkenny and Robinson 2018). We initiated enhancer identification across the genome in cell lines with existing data: A549, HepG2, K562, and MCF-7. We defined enhancers using assays independent of the training data to prevent circularity. Although various genomic assays exist for identifying potential enhancers, we used STARR-seq and ATAC-seq datasets to establish our initial set of enhancers for network training.

STARR-seq, a hybrid *in vitro*/*in vivo* assay, evaluates enhancer activity by assaying the capability of a given DNA sequence to drive an expression construct within a cellular context. The resulting data lack a true *in vivo* chromatin context due to the nature of the DNA that is assayed (i.e. genome-tiling plasmid constructs) (Arnold *et al.* 2013). STARR-seq peak calling was performed on all STARR-seq datasets with control samples using the STARRPeaker processing pipeline and peak caller (Lee *et al.* 2020). The STARRpeaker-provided hg38 covariate reference files and blacklist (ENCFF419RSJ) were used to call two sets of peaks. One set used a more stringent threshold (*P *< .05), and the other used a more lenient threshold (*P* < .1). Control samples were matched according to information in the ENCODE portal.

ATAC-seq identifies regions of open chromatin throughout the genome (Buenrostro *et al.* 2013). ATAC-seq derived peaks of open chromatin have previously been demonstrated to be enriched for biochemically active regions of the genome including enhancers (Yan *et al.* 2020). ATAC-seq narrowPeak coordinate files from each of the four cell lines were used to mark regions of accessible chromatin. These were produced by ENCODE’s standard processing pipeline that merged replicates from samples with matching cell lines, called peaks, and applied an exclusion list regions filter (ENCODE 2012). Overlapping peaks within each file were merged into single peaks using BEDTools’ “merge” command (Quinlan and Hall 2010).

**Figure 1.**
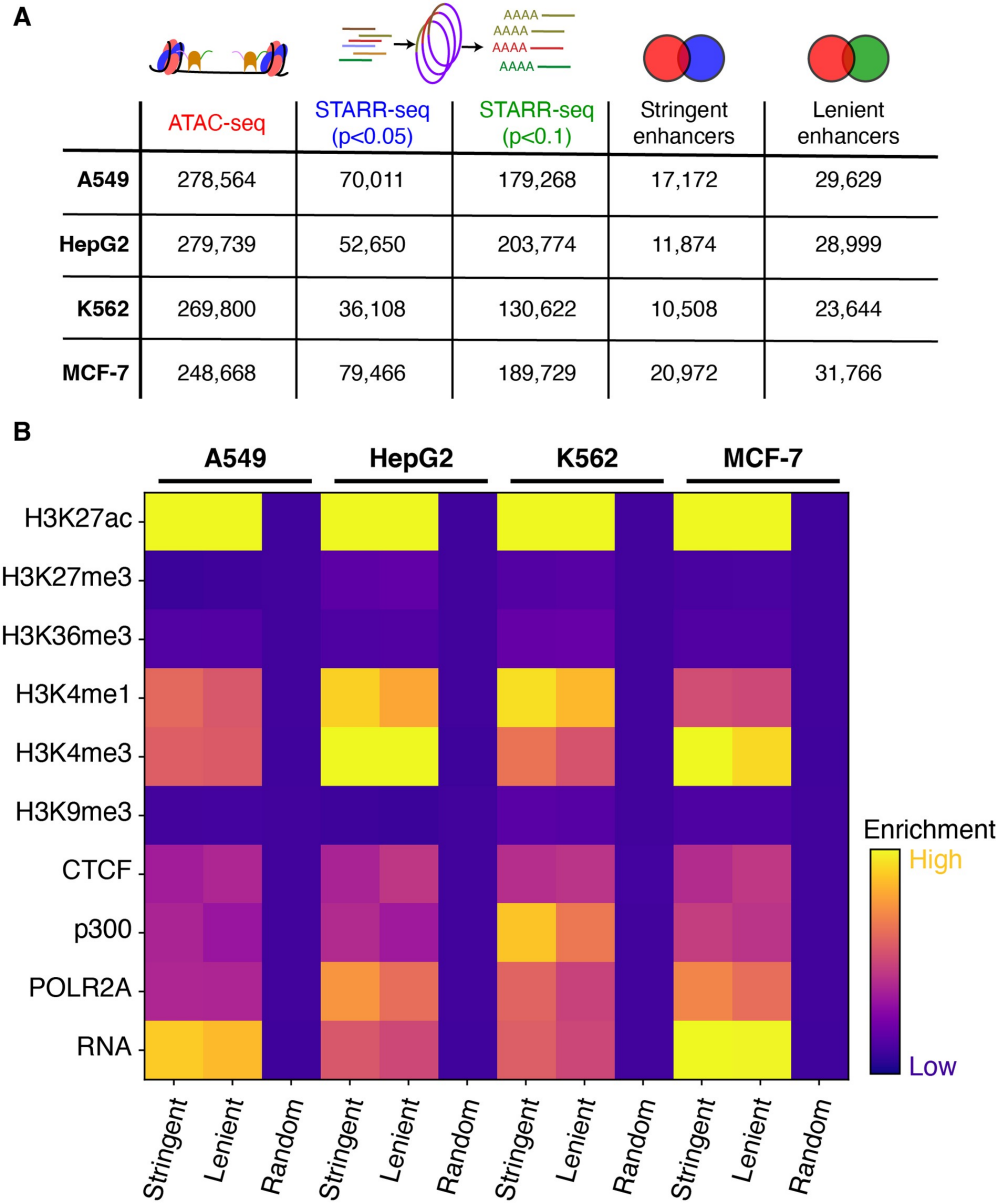
Overview of training data generation. (A) The training set of enhancers were generated by intersecting ATAC-seq peaks with STARR-seq peaks at *P* < .05 and *P* < .1 to generate “stringent” and “lenient” enhancer calls. (B) ENCODE ChIP-seq and nascent RNA run-on reads were summed in 1 kb windows centered on stringent enhancers, lenient enhancers, and 20 000 random genomic loci.

We called putative enhancers as the intersection of STARR-seq peaks and ATAC-seq peaks. Two separate lists of enhancers were generated by modulating the stringency of the STARR-seq peak-caller (STARRpeaker) to generate lenient and stringent enhancers (Fig. 1A). This created a list of genomic regions that contained DNA capable of enhancer activity (STARR-seq) and were known to be biochemically active in that genome (ATAC-seq). Each enhancer window was resized to a 1 kb length window. This resulted in eight sets of enhancers: “stringent” enhancer calls across each of the four cell lines and “lenient” enhancer calls across each of the four cell lines (4 cell lines × 2 types of enhancers). Each enhancer call was resized from the midpoint to a total 1 kb window.

**Figure 2.**
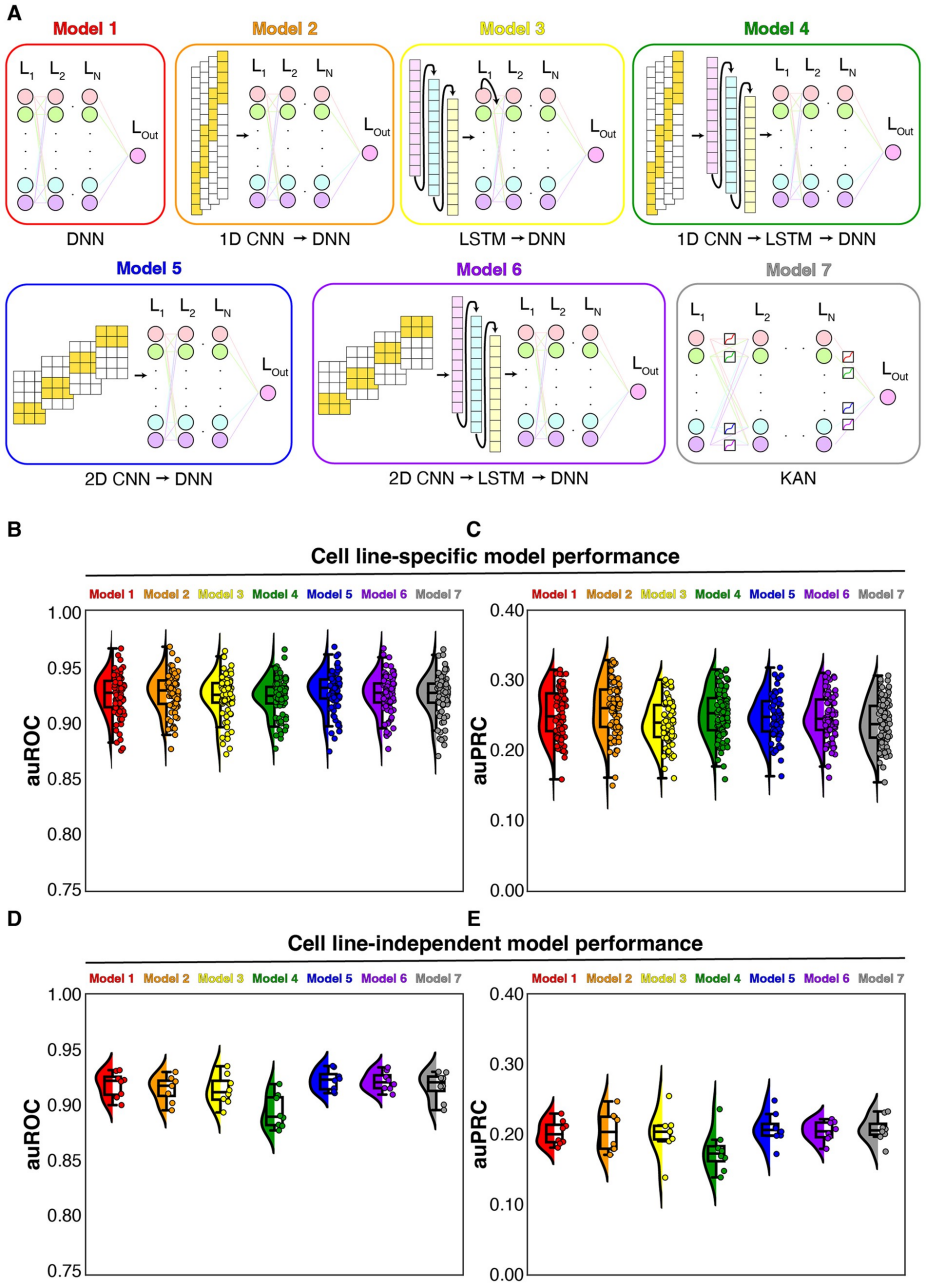
Performance of model architectures. (A) Cartoon overview of each model architecture. (B) auROC box-whisker and violin plot for cell line-specific architectures. (C) auPRC box-whisker and violin plot for cell line-specific architectures. (D) auROC box-whisker and violin plot for cell line-independent architectures. (E) auPRC box-whisker and violin plot for cell line-independent architectures.

We validated these enhancers by measuring the enrichment of histone modifications, transcription factors, and nascent RNA at these sites compared to random genome loci (Fig. 1B). Both lenient and stringent sets of predicted enhancer coordinates showed significant enrichment for classic markers of active enhancers such as H3K27ac and H3K4me while showing minimal enrichment for heterochromatin markers H3K27me3 and H3K9me3 (Creyghton *et al.* 2010, Zenk *et al.* 2017, Local *et al.* 2018, Methot *et al.* 2021). We also saw an enrichment for p300, a histone acetyltransferase used as a proxy for enhancers, and strong non-coding nascent transcription, previously shown to be a marker of enhancer activity (Tippens *et al.* 2020). We checked our predictions using an alternative set of predicted cell-line specific enhancers (chromHMM) to confirm that our predicted enhancer set was enriched for known enhancer chromatin signatures (Fig. 1, available as supplementary data at *Bioinformatics* online) (Ernst and Kellis 2012). Consistent with previous approaches, we also found that our enhancer training set was highly cell type specific, with the vast majority of called enhancers unique to each cell line (ENCODE 2012) (Fig. 2, available as supplementary data at *Bioinformatics* online).

### 2.3 Training and hold-out dataset generation

Hold-out data were generated using two different procedures: chromosomal hold-out procedure and cell line hold-out. Both procedures start by first tiling the genome into non-overlapping 1 kb windows and then filtering the windows to exclude blacklisted regions (ENCFF356LFX). For the chromosome hold-out, 1 kb bins spanning: chr7, chr8, chr9, chr10, chr11, chr12, chr13, chr15, and chr16 were generated. Bins were subsequently labeled as enhancer (intersect at least 25% with enhancer call) or else as a non-enhancer. In the cell line hold-out procedure, a similar process was performed but for the entirety of the hg38 tiled genome.

Training data were generated using two similar procedures for the chromosome hold-out set and the cell line hold-out set. For the chromosome hold-out training data, two hold-out chromosomes were removed from the genome, and the remaining bins were filtered to be at least 25% overlapping with an enhancer label. The enhancer bins were then boosted by duplicating each row 10 times with a 20 bp coordinate shift for a total window of 200 bp. Negative controls were calculated as bins 10 kb upstream and downstream from the positive bins to include negative sites with a similar local chromatin background. A set of random coordinates equal to the number of positive sites and not overlapping a predicted enhancer were also added as negative sites. Coordinate order was randomized to remove input bias. This procedure was also performed for each of the four cell lines independently. The four cell lines were permutated in combinations of 3, with the training data for those three cell lines concatenated together create the training data for the cell line networks.

For all hold-out and training dataset reference coordinate sets, ChIP-seq tag occupancy was calculated using the ScriptManager analysis platform at 10 bp resolution for a total vector size of 100 for each bin (Lang *et al.* 2022). Data were compressed into a hdf5 dictionary for rapid access for network training and performance evaluation.

### 2.4 Model architectures

The experimental setup involved evaluating seven deep-learning model architectures, designated as models 1–7 (Fig. 2A).

**Model 1:** Deep neural network (DNN) with an input dimensionality of 500 and a single output unit. It features three fully connected hidden layers, each with 256 nodes. Rectified Linear Unit (ReLU) activation functions are applied after each hidden layer to introduce non-linearity. To address overfitting, dropout layers with a probability of 0.5 are incorporated following each ReLU activation. The final output layer utilizes a sigmoid activation function to convert the network’s output to [0,1].

**Model 2:** Convolutional neural network (CNN) linked to a DNN. The CNN component consists of a one-dimensional convolutional layer, followed by a ReLU activation function and a max-pooling operation. There were three CNN layers of 16, 32, and 64. This design enables the network to capture spatial hierarchies and local patterns within the input chromatin data. The DNN component replicates the structure of model 1.

**Model 3:** Long short-term memory (LSTM) architecture with a hidden layer size of 500 and three LSTM layers. The output from the LSTM layers is then processed by a model 1 DNN.

**Model 4:** CNN (model 2) linked to an LSTM (model 3) with a final DNN (model 1). The CNN extracts the features from the input data, which are then processed by LSTM layers to capture temporal dependencies. The output of the LSTM is fed into the DNN for regularization.

**Model 5:** 2D CNN linked to a DNN. There were two CNN layers of 16 and 32. The CNN layers capture 2D spatial hierarchies in the data, while the DNN layers perform classification based on the extracted features.

**Model 6:** 2D CNN (model 5) linked to an LSTM (model 3) with a final DNN (model 1).

**Model 7:** Kolmogorov–Arnold network (KAN). The KAN is architecturally similar to the multi-layer perceptron except learnable activation edges have replaces the static weights connecting each node in the network. The KAN we implemented is based on the efficient-KAN implementation in PyTorch and was composed of four layers of 256 ending in a sigmoid function for binary classification (https://github.com/Blealtan/efficient-kan).

### 2.5 Network training

All networks were trained on NCSA’s Delta HPC. The chromosome hold-out networks were trained on either a ½ (models 5–7) or ¼ (models 1–4) A100. The cell line hold-out networks were all trained on a ½ A100. All large (330) networks were trained on a full A100.

### 2.6 Network-based enhancer cross-comparison

Predicted enhancers were called for each of the trained networks across each model architecture and cell line hold-out set. Each replicate of the held-out cell line was run through the network to generate a 0 to 1 prediction score for every genomic tile. This was repeated across all networks to generate two replicates each from 28 trained networks (7 model architectures × 4 cell line hold-out datasets) for a total of 56 genome-wide prediction score sets. A correlation matrix was generated for each pairwise genome-wide prediction scores and hierarchically clustered.

The top 1% of enhancer scores for each cell line replicate were intersected using “bedtools intersect” to create a set of conserved enhancer calls. The output from the conserved cell line enhancer calls was then intersected between each cell line and labeled. The four-way data labels were visualized by a python script implementing the venny4py package.

### 2.7 Predicted enhancer overlap with ENCODE features

A total of 1449 ENCODE ChIP-seq peak calls were downloaded from the ENCODE portal and bedtools intersected with each set of conserved cell-line specific enhancer calls. As a control, 25K “peaks” 1000 bp wide were randomly sampled from the genome and bedtools intersected with each conserved cell line-specific enhancer set. The Poisson means test with Benjamini–Yekutieli correction was calculated for each set of intersections. A corrected threshold of 0.01 was used to determine enrichment for each cell line enhancer set across each ENCODE peak set.

ChIA-PET data were downloaded from the ENCODE portal. Known canonical genes were downloaded from the UCSC Table browser, and a 1 kb window was centered on each gene’s start to define gene promoters. Each conserved cell line enhancer set and 25K random genomic loci were intersected with the ChIA-PET data and gene promoters. ChIA-PET entries that intersected with one enhancer and one promoter were tabulated for both enhancers and random loci. A log_2_ ratio was calculated as the log_2_ ratio of Enhancer-Promoter contacts over Random-Promoter contacts.

### 2.8 XAI implementation

Saliency mapping, integrated gradient, and deepliftSHAP were performed using the Captum implementation (Kokhlikyan *et al.* 2020). APSO was performed on all trained networks using the APSO implementation we previously reported (https://github.com/EpiGenomicsCode/Adversarial_Observation) (Gafur *et al.* 2024). APSO was run with a sparsity of 0.999 to simulate the nature of ChIP data. Data vectors in the swarm were bounded to [0,1] to mimic the normalization strategy of the ChIP data used to train the networks. Twenty epochs was sufficient to converge to a final vector across 500 particles for the cell line hold-out analysis. For the large network analysis, 100 epochs was used due to the substantially larger search space as a function of the larger training dataset.

## 3 Results

### 3.1 Model performance

We systematically evaluated various deep-learning classification architectures to determine the optimal network for predicting enhancers using only ChIP-seq data for eight different targets. These targets were selected to include a range of modified histones that mark enhancers (H3K4me1 and H3K27ac), promoters (H3K4me3), gene bodies (H3K36me3), and transcriptional repression (H3K27me3). We also included p300 that acetylates histone tails to mark histones like H3K27ac, Pol II which marks transcriptional activity, and CTCF, an architectural protein that binds chromatin and serves as a barrier to insulate regions of active and repressive chromatin (Mikkelsen *et al.* 2007, Cuddapah *et al.* 2009, Creyghton *et al.* 2010, Zentner *et al.* 2011, Local *et al.* 2018, Narita *et al.* 2021). These targets were selected to represent a minimal selection of known enhancer-related proteins and histone modifications which are present in the vast majority of human cell types. Our study included permutations of seven distinct architectures: DNNs, 1-D CNNs, LSTM, 2-D CNNs, and KANs. These network architectures were selected to represent the diverse range of the general architectures previously used for inference in genomics as well as the first usage of the KAN architecture for genomic inference to our knowledge (Srivastava *et al.* 2021, Talukder *et al.* 2021, Zhang *et al.* 2021, Alharbi and Rashid 2022).

We initially assessed the enhancer prediction capability of our seven network architectures. Each network was trained to predict enhancers using data from a single cell line (e.g. K562, HepG2, A549, MCF-7) with the final layer applying a sigmoid function that produced a confidence probability for each genomic locus. We reserved a rotating set of chromosomes from the training data for independent evaluation using stringent enhancer labels. Network performance was measured by calculating auROC and auPRC based on the networks’ accuracy in predicting enhancers in the held-out chromosomes, ranking each 1 kb window (Fig. 2B and C). No significant difference in performance was observed among the tested model architectures.

**Figure 3.**
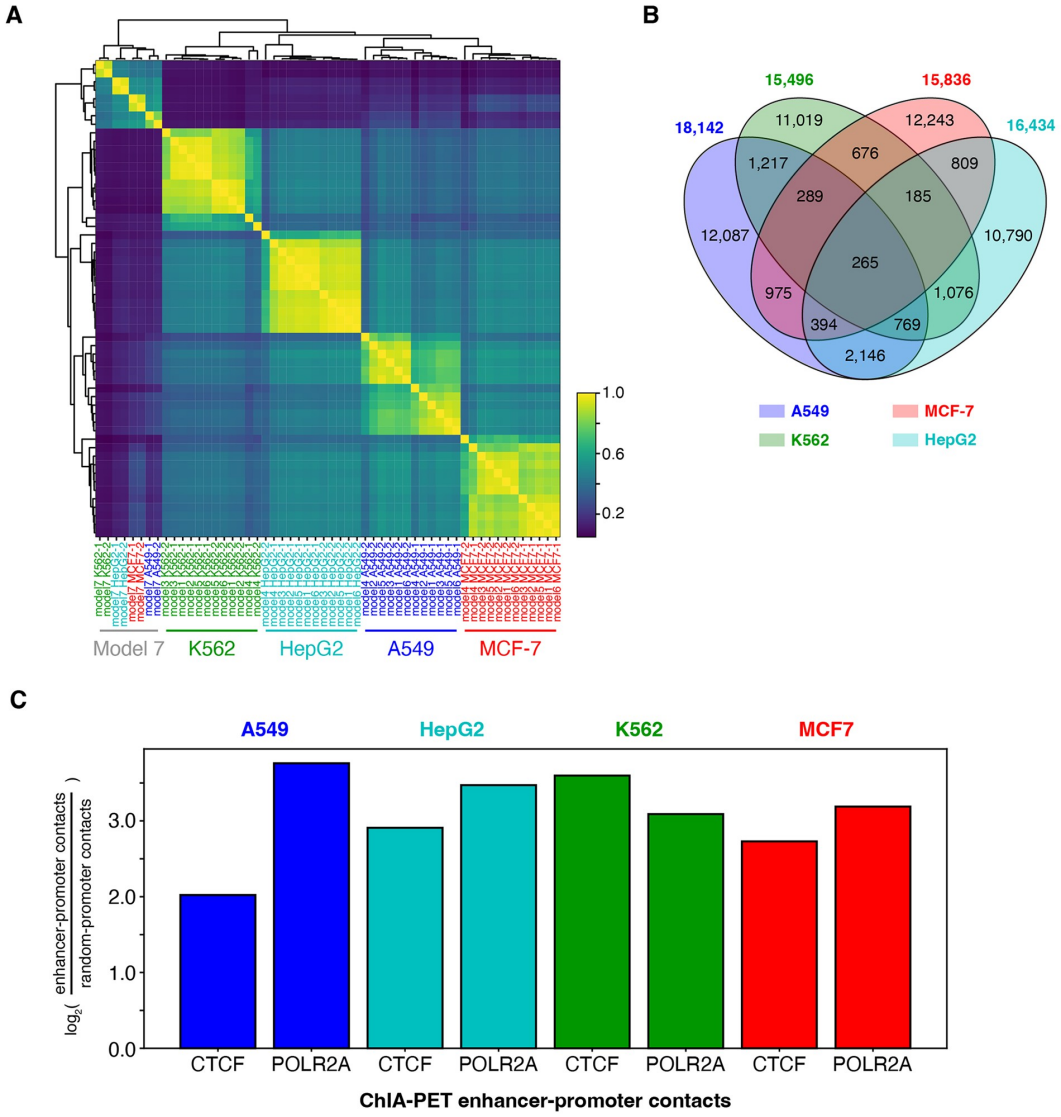
Architecture-specific enhancer comparison. (A) Whole-genome enhancer prediction scores were calculated across all cell lines on their respective hold-out cell line for each model architecture. Pair-wise hierarchical clustering of the enhancer correlation matrix was performed. (B) 4-Way Venn diagram intersect of the best performing model 5 cell line enhancers support the high amount of cell line specificity on our predicted enhancers. (C) ChIA-PET 3D contacts for enhancer-promoter contact relative to random loci-promoter contacts.

Subsequently, we evaluated the models’ ability to predict enhancers across different cell lines. We combined data from three of the four cell lines for training while using the remaining cell line for validation. As anticipated, auROC and auPRC decreased compared to the cell-line-specific models due to the increased complexity of the training task with the same data dimensionality. Notably, model 4 (1D CNN—LSTM) exhibited a more pronounced decrease in auROC and auPRC compared to the other models (Fig. 2D and E). In contrast, model 5 (2D CNN) performed the best with a median auROC and auPRC of 0.924 and 0.206, respectively. Although we note that besides these minor differences in auROC and auPRC, there are minimal performance differences between the model architectures. We also calculated the average loss per training epoch for each model trained and confirmed that 20 epochs was sufficient to plateau accuracy loss (Fig. 3, available as supplementary data at *Bioinformatics* online).

### 3.2 Comparison of architecture-derived enhancers

Evaluating a model’s performance extends beyond summarizing results with metrics such as auROC and auPRC and include biological verification that the predictions are relevant in a genomic context. To assess the reproducibility and biological relevance of predicted enhancers, we computed genome-wide enhancer scores for every 1 kb window across the human genome using each of the models on their respective held-out cell line. We then calculated the pairwise correlation of each prediction vectors and performed 2D hierarchical clustering on the resulting correlation matrix (Fig. 3A). Notably, pair-wise correlations within each cell line generally dominated the clustering independent of model architectures with one exception. This implied that a sizeable fraction of enhancers predicted by our networks are cell type specific.

**Figure 4.**
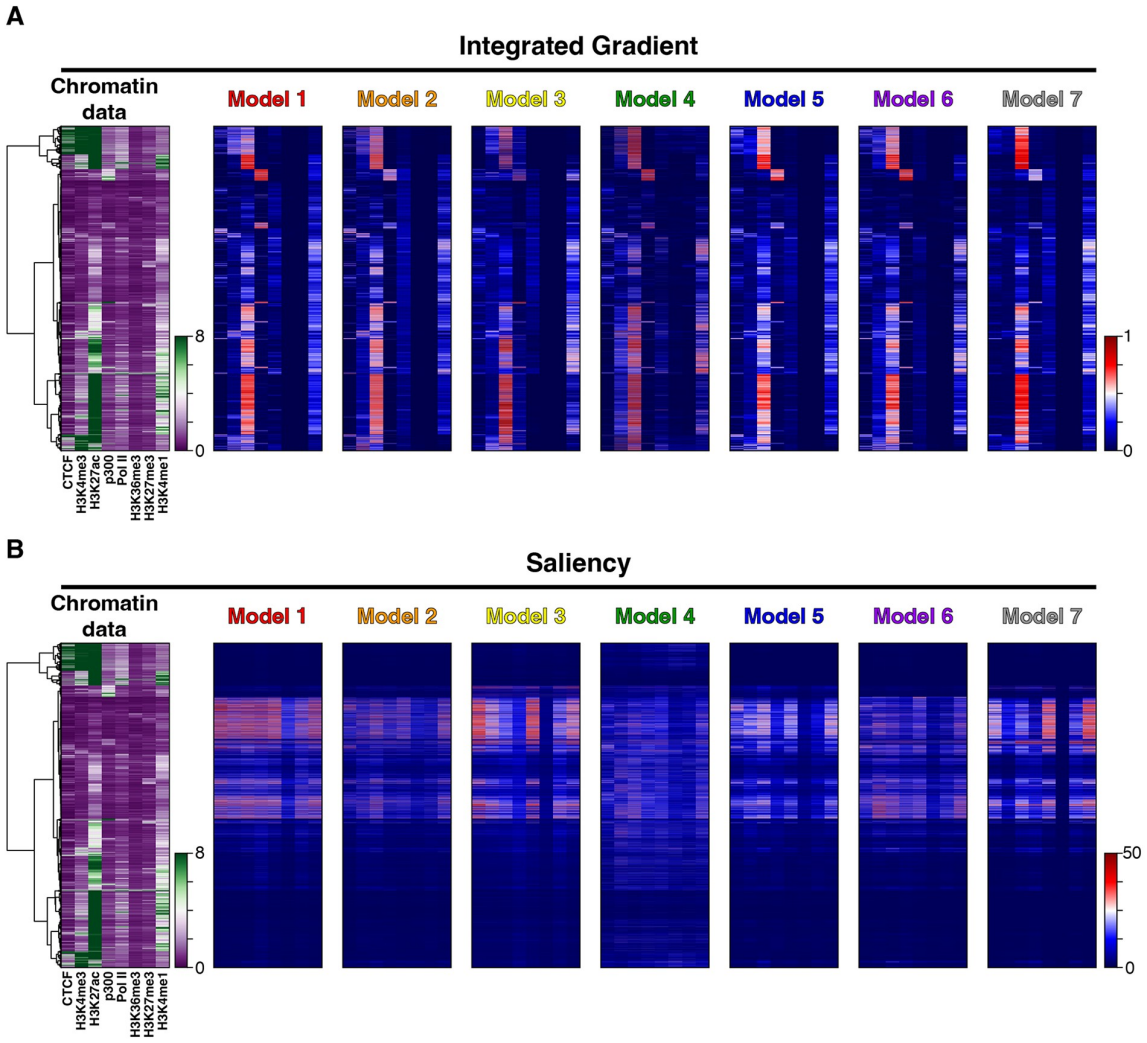
XAI analysis of MCF-7. (A) The held out MCF-7 cell line was filtered to only contain enhancer labels and fed into integrated gradient calculations. Hierarchical clustering was performed on the ChIP occupancy data and then binned to a single bin for visualization purposes. The integrated gradient score was high across all models for H3K27ac, p300, and H3K4me1, indicating they were primarily used for inference and others features such as CTCF and H3K4me3 were generally not used even when present in the vector. (B) Saliency maps were calculated and generated for all models following the above procedure. Saliency heatmaps show that the networks leverage additional data when the vectors are sparse, except for H3K36me3.

We took the top 1% set of our rank-sorted whole genome tiles for each cell line and examined the intersection across different models using distinct biological replicates. This analysis confirmed the clustering pattern observed in the correlation matrix, revealing that most genomic regions with predicted enhancer identities were specific to the individual cell-type (Fig. 3B). The exception to this was enhancers predicted using the KAN architecture, which showed consistent correlations across cell lines irrespective of cell line identity. We found that KAN-predicted enhancers were just as likely to be cell line specific as enhancers predicted by the other architectures (Fig. 4, available as supplementary data at *Bioinformatics* online).

A key feature of enhancer activity is their ability to regulate gene expression at a distance. This functionality is thought to be mediated through three-dimensional folding of DNA, which brings enhancers into close physical proximity to the target gene promoter. To investigate this, we examined the physical interactions of our predicted enhancers with gene promoters using the 3D enrichment assay ChIA-PET, focusing on CTCF and POLR2A (Fig. 3C). ChIA-PET for these factors has previously been demonstrated to enrich for enhancer elements (Tang *et al.* 2015). For each cell line’s predicted enhancers, we found our predicted enhancers to physically engage with gene promoters substantially above background.

We next examined the epigenomic characteristics of the predicted enhancers for each cell line. We performed a large-scale enrichment test of ENCODE-derived transcription factor ChIP-seq peaks at predicted enhancers for each cell line (Table 2, available as supplementary data at *Bioinformatics* online). We found substantial enrichment of known enhancer-associated transcription factors enriched at the predicted enhancers (i.e. FoxA1/2/3, CEBPA, YY1, etc.) and general activator transcription factors (i.e. SP-1) in support of these regions’ identity as enhancers (Pabst *et al.* 2001, Lupien *et al.* 2008, Weintraub *et al.* 2017, Fu *et al.* 2019, Hasegawa and Struhl 2021).

### 3.3 Interpretation of trained networks

A major challenge in utilizing neural networks for research is their “black box” nature, which complicates interpretation of how they reach conclusions. The field of XAI aims to address this issue by developing methods to reveal the features that influence network predictions. Given that our networks were predicting cell-type-specific enhancers with an orthogonal validation, we sought to understand how the eight datasets used for training influenced the predictions and whether the different architectures used different aspects of the input data. We used Captum’s implementation of integrated gradients, saliency, and SHAP to each model architecture. This allowed us to directly compare the contributions of each dataset and architecture combination to the predictions (Simonyan *et al.* 2013, Lundberg and Lee 2017, Shrikumar *et al.* 2017, Sundararajan *et al.* 2017, Kokhlikyan *et al.* 2020).

We used the best-performing dataset across all network architectures (K562-HepG2-A549 replicate 2) and applied integrated gradients and saliency techniques to analyze known enhancers from the hold-out cell line (MCF-7). We then linked the results of XAI methods to the original data and performed hierarchical clustering on the raw input data, aggregating 1 kb windows into single bins per feature for visualization. Integrated gradients highlighted a strong enrichment of H3K27ac as a key driver of inference, with p300 and H3K4me1 also contributing to the model’s predictions when present (Fig. 4A). The saliency map differed from the integrated gradients, revealing that in cases of sparse data, the network activated additional neurons to compensate for the absence of clear driver features (Fig. 4B). Overall, the results from deepliftSHAP closely mirrored those of integrated gradients, indicating substantial functional overlap between these techniques (Fig. 3, available as supplementary data at *Bioinformatics* online).

### 3.4 APSO identifies patterns of chromatin binding proteins predictive of enhancer status

**Figure 5.**
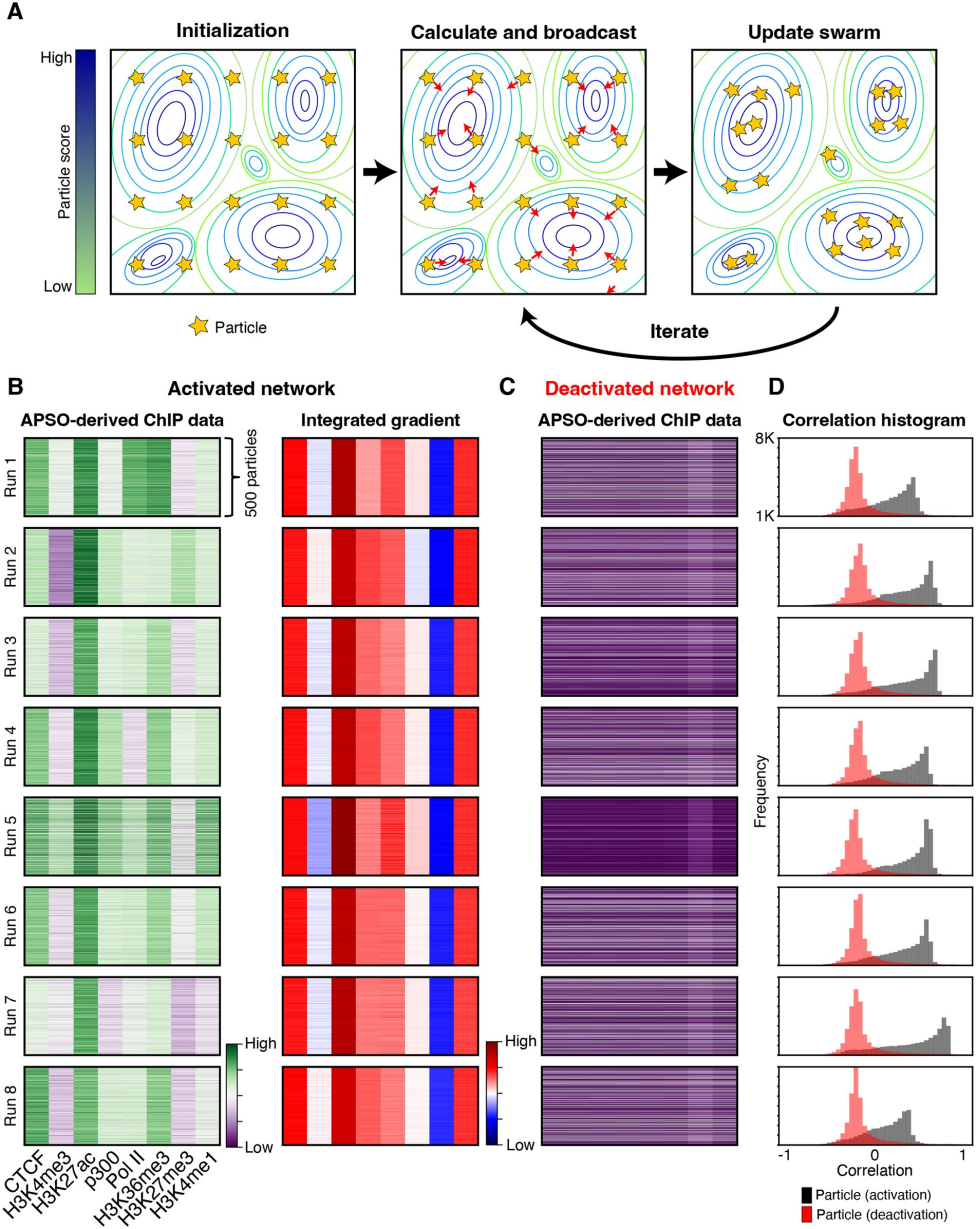
APSO attack of model 5. (A) APSO simulates a swarm of particles, where each particle represents a vector of input data. The particles explore numerical space, guided by their own best-known positions and the swarm’s overall best position to minimize the loss function. (B) Eight iterations of APSO with 500 particles for 20 epochs were performed with the best scoring model 5 to generate synthetic ChIP-seq data. Integrated gradients were calculated using APSO-derived data. All data were binned to 1 bin per feature for visualization. (C) The cost function of APSO was inverted to optimize for negative features and run for eight epochs. (D) The average profile of the positive and negative APSO chromatin profiles was correlated against the hold-out MCF7 enhancers to generate a frequency histogram of the correlation scores.

A key challenge for many XAI techniques is the need for hold-out data to identify the specific features that activate a trained model. This requirement can be problematic when using pre-trained models from the community, as hold-out data are often a subset of the training data and may include the same artifacts present in the full dataset. As hold-out data are typically a subset of the training data, it naturally will include any of the technical or biochemical artifacts that were present in the full dataset. We recently developed a XAI technique with a foundation in “adversarial particle swam optimization” (APSO) that can decipher features of interest in trained networks in an architecture-agnostic manner and without any preconceptions about the overall structure of the training data (Gafur *et al.* 2024). APSO operates by initializing a “swarm” of random vectors of data (i.e. particles) from a uniform distribution in the same shape as the training data and feeding those vectors into a trained network. The randomly initialized particles are scored according to their ability to predict an enhancer and the information contained withing the best scoring particle is broadcast to the entire swarm. This process iterates for a set number of epochs (Fig. 5A).

Given a set of n particles, a swarm can be calculated by:


(1) $$S=\{p_{1}\ldots p_{n}\}$$

A particle (p_i_) within the swam has velocity (v_i_) that is composed of inertia weight (w_i_), cognitive weight (c_1_), and social weight (c_2_). Inertia weight controls the impact of the previous velocity on the current velocity. The cognitive and social coefficients are scaled by random uniform values, r_1_ and r_2_, respectively. The cognitive weight is further scaled by the personal best historical location (h_i_), and the social weight is scaled by the current global best location (g_i_). The calculation of a particle’s velocity can be represented as:


(2) $$\vec{v_{i}}(t+1)=\omega \vec{v_{i}}(t)+c_{1}r_{1}(\vec{h_{i}}-\vec{r_{i}}(t))+c_{2}r_{2}(\vec{g}-\vec{r_{i}}(t))$$

For every epoch of APSO, each particle updates its current location based on its newly calculated velocity plus its previous location li. The update rule can be expressed as:


(3) $$\vec{l_{i}}(t+1)=\vec{l_{i}}(t)+\vec{v_{i}}(t+1)$$

We applied APSO to our best-performing network, which was based on the 2D CNN architecture. APSO, in the context of our networks, generates *in silico* ChIP-seq data at the same resolution and order of our training data. We analyzed this synthetically generated ChIP data generated across eight runs of the algorithm, along with calculating the corresponding integrated gradients (Fig. 5B). This approach successfully reproduced the chromatin patterns observed in the original training data, including high levels of H3K27ac and H3K4me1. Interestingly, we observed nearly identical patterns in the network’s integrated gradient calculations, regardless of variations in synthetic chromatin data. This suggests that like the overall performance across different architectures, the trained network rely on only a subset of the total chromatin data for making predictions.

We next used a unique feature of APSO, which is its ability to invert the cost-function that typically converges the particle swarm to positively predictive features and instead converged the swarm to negatively predictive features. In the context of our networks, this technique allows us to identify aspects of the ChIP-data that were strongly anti-predictive of enhancer activity. Applying this approach, we identified a chromatin signature representative of “not enhancer” as low signal for all datasets except for H3K27me3 (Fig. 5C). This finding strongly matches the known biology of H3K27me3 being a strong marker of repression and anti-correlated to enhancer activity (Cai *et al.* 2021).

To assess the biological validity of the synthetic chromatin data, we calculated the average chromatin score for each of the eight ChIP targets across the eight APSO runs and compared the average profile’s correlation against the held-out MCF-7 enhancer data. We also computed the correlation for the deactivated APSO chromatin profiles against the held-out data. A frequency histogram was constructed to compare the correlation frequencies between the activated and deactivated APSO chromatin profiles (Fig. 5D). The results indicated that the activated network profiles, with a strong average correlation mode of 0.6, were more representative of biological data relative to the deactivated network profiles, which had a mode correlation score of −0.2.

### 3.5 Large scale identification of combinatorial proteins predictive of enhancers

**Figure 6.**
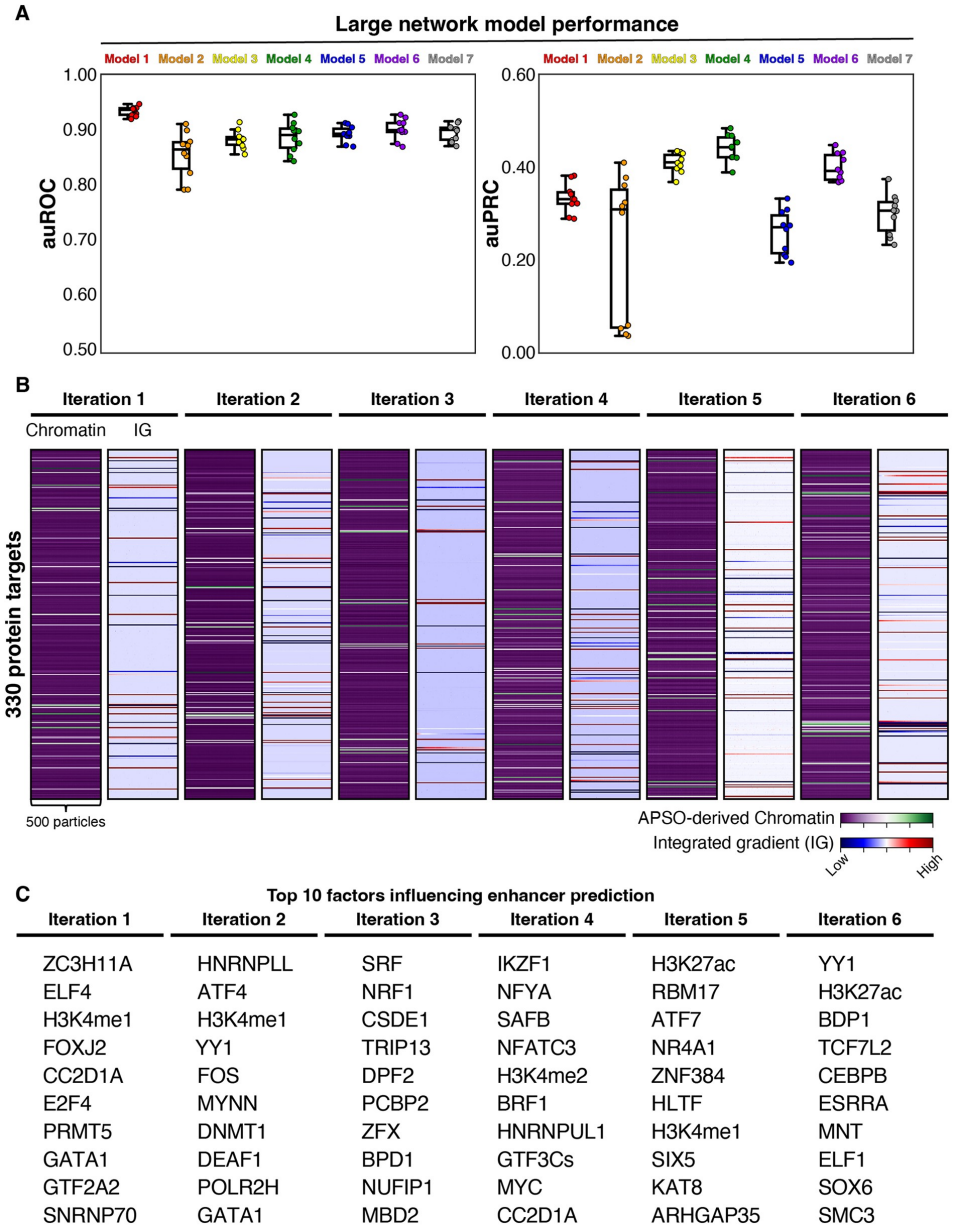
Large network performance and APSO results. (A) auROC and auPRC box and whisker plots for each model after chromosome hold-out training using 330 ChIP-seq datasets. (B) APSO of the best performing model 4 for 100 epoch and 6 iterations. Integrated gradient was calculated using the synthetic ChIP data to identify activation weights for each dataset. Data were summed to 1 per feature for visualization. (C) Top 10 features per APSO iteration (synthetic ChIP data * integrated gradient).

Given the success of APSO in identifying predictive features of chromatin performance for enhancer inference, we then examined how well APSO scaled for networks trained on larger datasets. We processed 330 ENCODE-derived K562 ChIP-seq datasets through our chromosome hold-out process described above and tested each of the seven model architectures (Fig. 6A). Surprisingly, model 4 (1D CNN—LSTM—DNN) which had previously performed the worst in the cell line independent training, now had the highest average auPRC of all models. Strikingly, model 2 (LSTM—DNN) had negligible auPRC scores in almost half the models trained using the large dataset. This likely reflects the known attention-limitations of the LSTM architecture in cases where the training dataset is large and suggests this architecture is unstable for networks trained on large amounts of ChIP-seq data (Hochreiter and Schmidhuber 1997).

We applied APSO to the best performing (by auPRC) large-scale network to generate *in silico* chromatin datasets and calculated the corresponding integrated gradient. Due to the size of the network, computing the integrated gradient across the entire hold-out dataset was computationally prohibitive. APSO, being a primarily CPU-oriented algorithm, is extremely scalable compared to the Captum’s implementation of integrated gradient. Across six runs of APSO for 100 epochs, we identified six completely distinct profiles of chromatin datasets that were predictive of enhancer activity (Fig. 6B). The rank-order sort of the *in silico* datasets normalized by their integrated gradient score was used to identify the protein factors driving enhancer prediction within our network. The correlation of normalized contribution scores across eight runs was near 0, indicating each run of APSO on the large network largely generated completely distinct lists of proteins predictive of enhancers (Table 3, available as supplementary data at *Bioinformatics* online). Supporting this, the top predicted proteins across all APSO runs were highly unique relative to each other, indicating that the network had learned a diverse set of combinatorial rules for its inference making (Fig. 6C). In addition to identifying the same factors (H3K4me1, H3K27ac) we used in the limited training, APSO also identified many previously characterized enhancer-related proteins including GATA1, MYC, YY1, and many others. GATA1 in particular is known to be a cell-type-specific sequence specific transcription factor enriched in K562 enhancers (Horak *et al.* 2002). Intriguingly, APSO also identified several RNA-splicing related factors such as SNRNP70, HNRNPLL, and HNRNPUL1 that have not been previously associated with enhancer activity, although RNA-splicing in general has been previously implicated in enhancer regulation (Sartorelli and Lauberth 2020).

## 4 Discussion

We conducted a thorough evaluation of various deep-learning network architectures for predicting enhancers using only a minimal set of chromatin datasets. Our analysis revealed that, given the information density of the datasets, the choice of architecture had minimal impact on the overall predictive performance. In most cases, a multi-layer DNN was adequate for achieving high accuracy and precision in enhancer prediction. Additionally, we observed no significant improvement in enhancer prediction using the KAN architecture. In fact, the enhancer predictions generated by KAN did not align well with those produced by other architectures. This finding is in line with a recent work demonstrating that the KAN architecture may be inferior to standard deep-network architectures in general use (Yu *et al.* 2024).

Our usage of XAI algorithmic approaches (i.e. integrated gradient and deepliftSHAP) confirmed that across all architectures, the same features (i.e. H3K27ac, H3K4me1) were used predominately for enhancer prediction. Applying APSO to our trained networks, we cross-validated the XAI findings of H3K27ac and H3K4me1 importance for enhancer inference while simultaneously validating APSO’s ability to re-construct ChIP-profiles agnostic to model architecture. The model-agnostic nature of APSO allows it to be broadly applied to any predictive framework to reconstruct the features that drive inference. Additionally, inverting the cost-function of APSO allows it to capture features that offer negative value, as seen in the “de-enrichment” of H3K27me3 for enhancer activity. The CPU-nature and low memory requirements of APSO lend itself to high scalability given the large of amount of cheap CPU compute available.

Previous genome annotation approaches typically use a binarization-style (i.e. enhancer/not-enhancer) of the genome and then examine data at segmented regions of interest (Ernst and Kellis 2012, Hoffman *et al.* 2012). However, this approach often focuses on datasets strongly enriched at loci of interest and can neglect targets that may appear “marginal” on their own. APSO excels in identifying combinatorial features that alone have poor predictive value. Our APSO approach on a large neural network trained on 330 unique ChIP datasets successfully generated multiple distinct feature profiles related to enhancer activity. The highly diverse results across APSO runs on the large network indicated a highly complex network without any single group of proteins solely driving enhancer prediction. Additionally, while each run enriched for previously characterized enhancer-related proteins, APSO also identified numerous proteins with no previously known direct-connection to enhancer biology. These results support the idea that APSO can be used as a discovery platform on sufficiently complex networks to identify potentially novel proteins related to enhancer activity. In the future, we envision using APSO to guide experimental decision making and investigate the combinational predictions it makes.

We also demonstrated that chromatin data (ChIP-seq) represented as numerical vectors of occupancy are sufficient to predict enhancers without the parallel need for DNA sequence content in the training data. Of course, DNA sequence is indirectly encoded as a function of sequence alignment to the reference genome; however, there are potential approaches that can remove the dependence of sequence alignment to a reference genome including popular pseudo-alignment approaches (e.g. kallisto) (Bray *et al.* 2016). Kallisto has already been adapted for scATAC-seq and could be further integrated with our enhancer prediction approach to completely remove reference genome dependency from enhancer prediction (Giansanti *et al.* 2020). Given the high prevalence of DNA sequence mutations in the patient-derived samples, approaches that side-step this requirement are needed to continue the advancement of AI into clinical applications.

## Supplementary Material

btaf371_Supplementary_Data
