# Supplementary figures and images for: Adversarial attack of sequence-free enhancer prediction identifies chromatin architecture
Source: Bioinformatics. 2025 Jun 24;41(7):btaf371. doi: 10.1093/bioinformatics/btaf371 (PMC12240468; doi:10.1093/bioinformatics/btaf371)

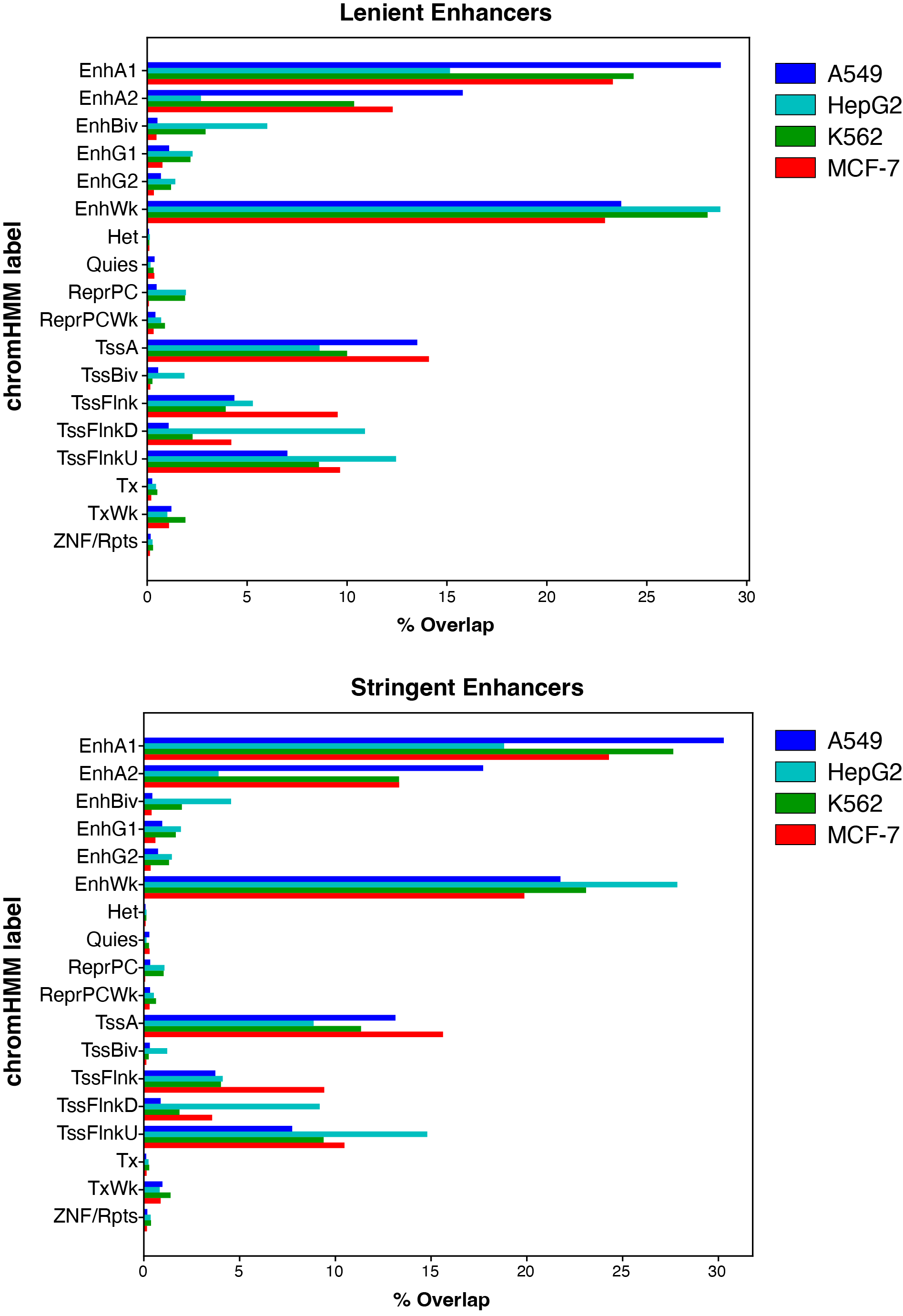

Supplement: btaf371_Supplementary_Data [file btaf371_supplementary_data.zip › SFigure1.png]

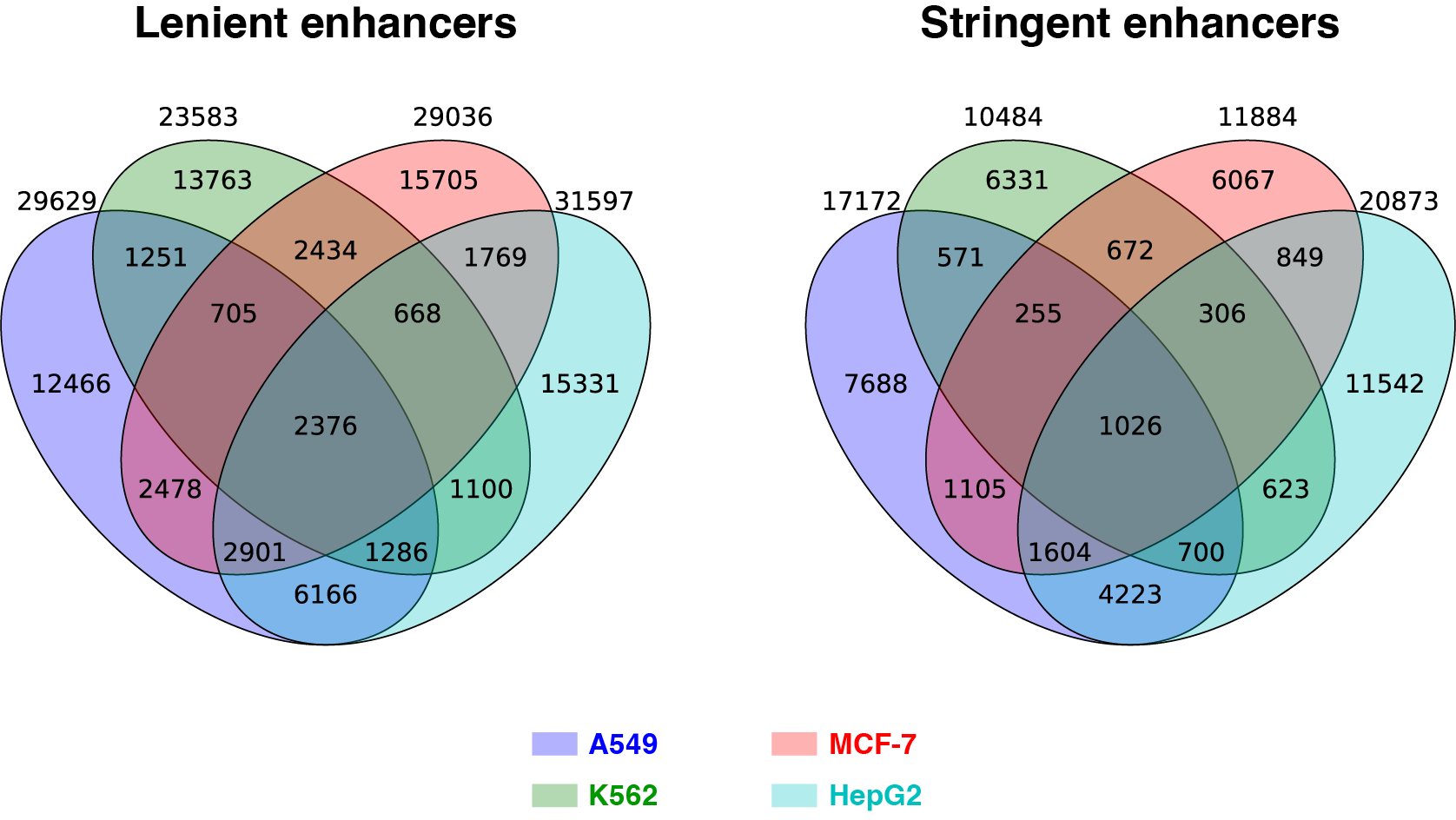

Supplement: btaf371_Supplementary_Data [file btaf371_supplementary_data.zip › SFigure2.png]

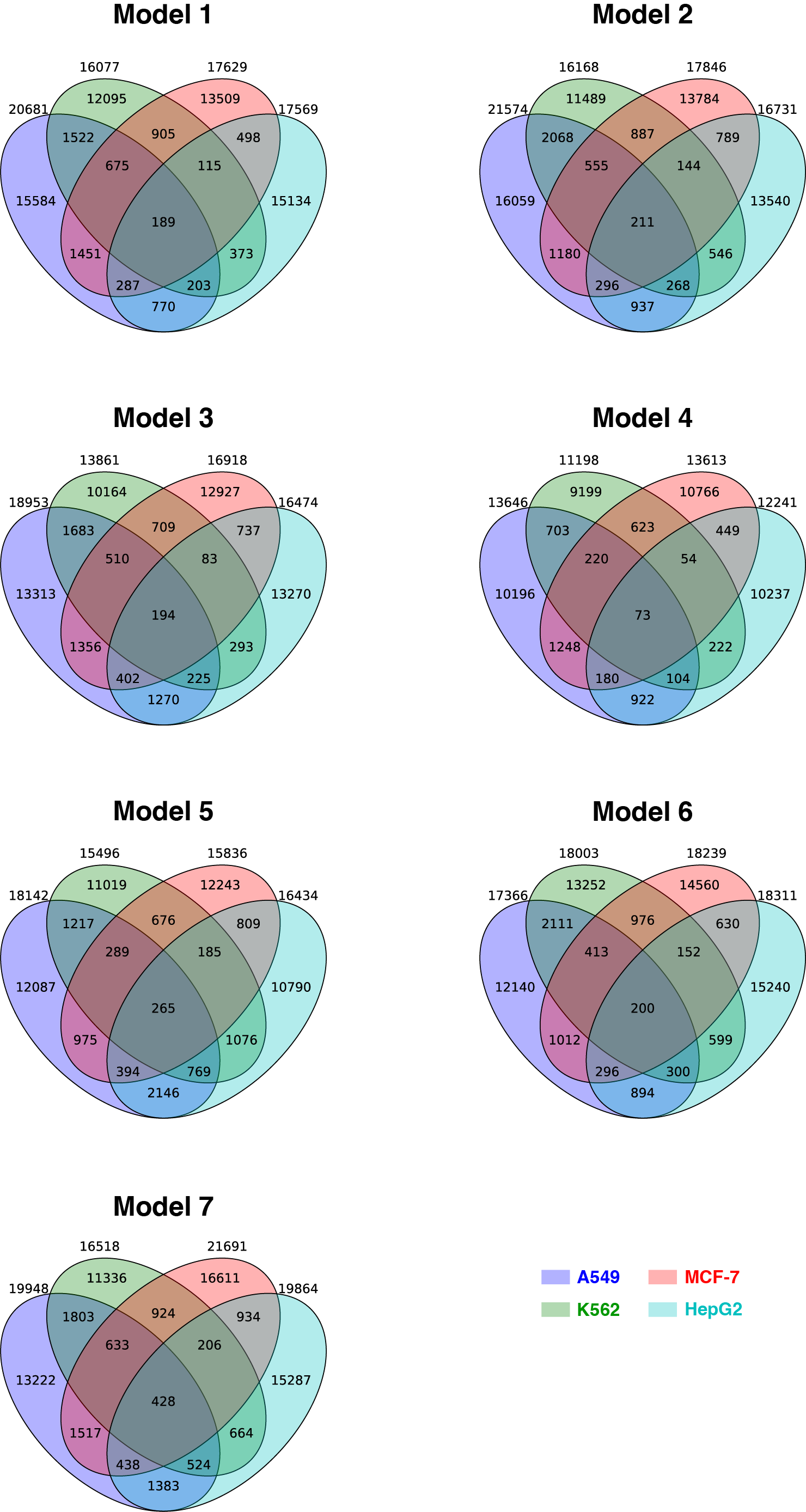

Supplement: btaf371_Supplementary_Data [file btaf371_supplementary_data.zip › SFigure4.png]

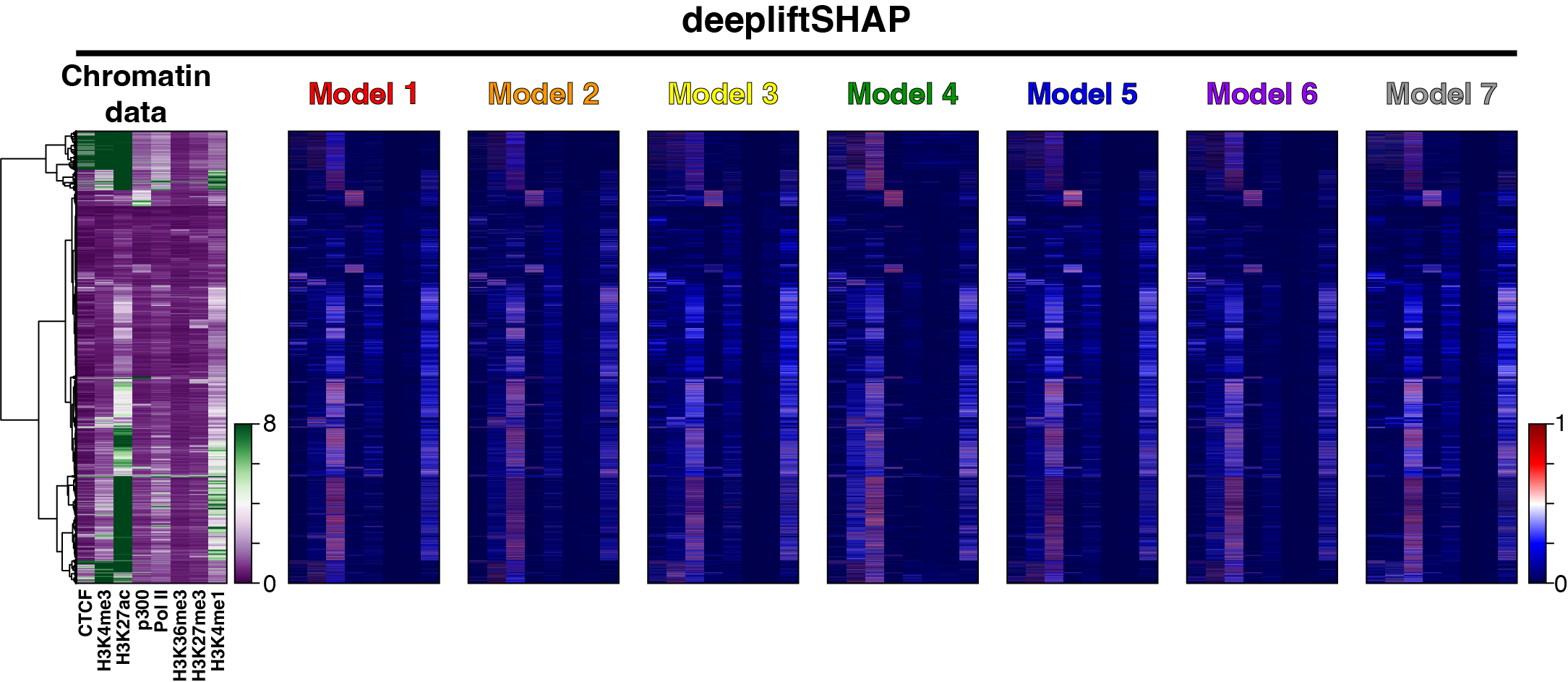

Supplement: btaf371_Supplementary_Data [file btaf371_supplementary_data.zip › SFigure5.png]

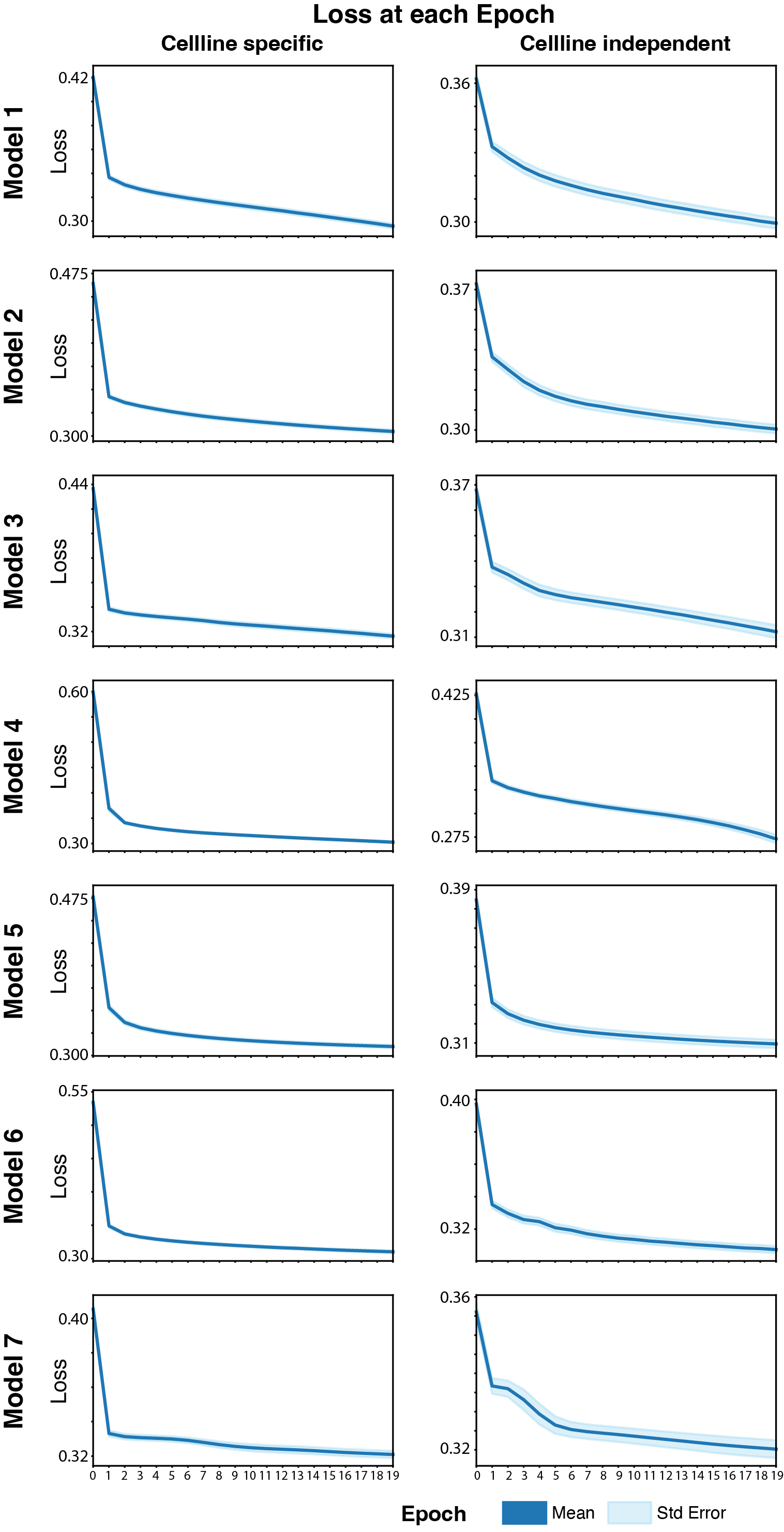

Supplement: btaf371_Supplementary_Data [file btaf371_supplementary_data.zip › SFigure3.png]
